# No-Touch Adaptive Versus Conventional Robot-Assisted Partial Nephrectomy for Localized Renal Tumours with High Nephrometry Complexity: A Comparative Analysis of Early Outcomes

**DOI:** 10.3390/cancers18101577

**Published:** 2026-05-12

**Authors:** Gianluca Giannarini, Alessandro Crestani, Giuliana Lista, Paolo Corsi, Gioacchino De Giorgi, Davide Minardi, Luca Di Gianfrancesco, Davide De Marchi, Antonio Amodeo, Angelo Porreca

**Affiliations:** 1 Urology Unit, Santa Maria Della Misericordia University Hospital, 33100 Udine, Italy; alessandro.crestani@uniud.it (A.C.); gioacchino.degiorgi@asufc.sanita.fvg.it (G.D.G.); davide.minardi@asufc.sanita.fvg.it (D.M.); 2 Department of Medicine (DMED), University of Udine, 33100 Udine, Italy; 3 Oncological Urology Unit, Veneto Institute of Oncology IOV-IRCCS, 35128 Padua, Italy; giuliana.lista@iov.veneto.it (G.L.); paolo.corsi@iov.veneto.it (P.C.); antonio.amodeo@iov.veneto.it (A.A.); 4 Urology Unit, Humanitas Gavazzeni, 24125 Bergamo, Italy; luca.digianfrancesco@gavazzeni.it (L.D.G.); davide.demarchi@gavazzeni.it (D.D.M.); angelo.porreca@hunimed.eu (A.P.); 5 Department of Biomedical Sciences, Humanitas University, Pieve Emanuele, 20072 Milan, Italy

**Keywords:** kidney cancer, renal cell carcinoma, renal function, robotic surgery, partial nephrectomy, enucleation, enucleoresection, renorrhaphy, ischemia, off-clamp, on-clamp, arterial clamping, complications

## Abstract

**Background/Objectives**: Surgical refinements in robot-assisted partial nephrectomy (RAPN) aim to reduce morbidity and optimize renal function preservation, particularly in patients with high-complexity renal tumours. This study describes the no-touch adaptive technique for RAPN and compares its perioperative outcomes, postoperative complications, and resulting early renal function with those of the conventional approach. **Methods**: A cohort of 72 consecutive patients with high-complexity renal tumours undergoing RAPN was evaluated. The study group included 38 patients treated with the no-touch adaptive technique, while 34 patients underwent the conventional approach. The no-touch adaptive technique consisted of sutureless, off-clamp, simple tumour enucleation with incremental haemostasis and the option to shift to arterial clamping, tumour enucleoresection, or renorrhaphy as needed. The conventional technique involved on-clamp minimal enucleoresection with double-layer renorrhaphy. Outcomes assessed included completion of a fully no-touch procedure, perioperative metrics, 90-day postoperative complications, and 3-month renal function change from baseline. **Results**: Baseline characteristics were comparable between groups. A fully no-touch RAPN was achieved in 30/38 (79%) patients. Adaptations were required in eight cases: shift to main arterial clamping (*n* = 2), renorrhaphy (*n* = 5), or both (*n* = 1), with one conversion to total nephrectomy due to intractable bleeding. Estimated blood loss was similar between groups (study: 150 mL [IQR 75–250] vs. control: 180 mL [IQR 100–400]). Length of stay was significantly shorter in the study group (3 days [IQR 3–4]) compared with controls (5 days [IQR 6–8]). Any-grade 90-day complications were significantly lower with the no-touch technique (21% vs. 56%, *p* < 0.01). Clinically significant 3-month eGFR decline occurred in 14% of controls versus 0% of study patients (*p* = 0.02). **Conclusions**: The no-touch adaptive RAPN technique is feasible in high-complexity renal tumours and provides reduced morbidity and superior early renal function preservation compared with the conventional approach.

## Simple Summary

This study compared two surgical methods for removing complex kidney tumours using robotic assistance while preserving as much healthy kidney as possible. The new “no-touch” technique avoids temporarily blocking blood flow to the kidney and aims to remove the tumour gently, using minimal stitches only when needed. Among 72 patients, those treated with this approach had fewer postoperative complications, recovered faster, and stayed in the hospital for a shorter time compared with those who underwent the traditional technique. Importantly, none of the patients in the no-touch group experienced a major drop in kidney function three months after surgery, while this occurred in 14% of patients treated conventionally. The findings suggest that the “no-touch” technique may offer a safer and more kidney-sparing option for people with complex kidney tumours, though larger studies are needed to confirm long-term benefits.

## 1. Introduction

Partial nephrectomy (PN) is recommended over radical nephrectomy (RN) in patients with clinically localized T1 tumours and in selected patients with T2 tumours and a solitary kidney or chronic kidney disease, if technically feasible [1]. However, treating tumours with high nephrometry complexity poses significant challenges, necessitating nuanced decision-making to carefully weigh the benefits and risks of PN versus RN [2,3]. When PN is pursued in such cases, clinicians must anticipate a higher risk of complications and a greater likelihood of renal function decline compared to treating less complex tumours [4].

In recent years, robot-assisted PN (RAPN) has gained substantial popularity over open PN, owing to advantages such as reduced blood loss, lower perioperative morbidity, and faster recovery, while maintaining comparable oncological outcomes [5,6]. This shift has been accompanied by a growing focus on optimizing functional outcomes, namely, minimizing morbidity, preserving renal function, and enhancing quality of life, primarily through refinements in surgical technique. However, despite being in practice for over two decades, RAPN still lacks a universally accepted standard for its procedural steps. Arterial inflow control, tumour excision technique, and parenchymal reconstruction can all significantly influence both perioperative and functional outcomes. Multiple technical variations for each surgical step, applied either individually or in combination, have been investigated, often yielding conflicting results. In addition, various strategies and emerging technologies have been proposed [7,8,9], yet the supporting evidence remains inconclusive. Consequently, current urological guidelines offer limited and inconsistent recommendations, a gap that is especially pronounced in the context of RAPN for patients with high-nephrometry-complexity tumours.

Over the past years, we have developed what we term no-touch adaptive RAPN, consisting of a sutureless off-clamp simple tumour enucleation with incremental haemostasis and on-demand shift to conventional technical steps such as arterial clamping, tumour enucleoresection and/or renorrhaphy, if needed. This approach serves a dual purpose: minimizing the vascular and parenchymal injury to the kidney, thereby maximizing renal function preservation, and reducing the risk of perioperative complications. We hypothesize that the greatest benefits of this technique, although employed universally in our practice, would be most evident in patients with high-nephrometry-complexity tumours, where the risks of morbidity and renal function decline are greatest.

Therefore, the objectives of this study were to describe our no-touch adaptive technique for RAPN, and to compare its perioperative outcomes, complication rates, and early impact on renal function with those of the conventional technique in patients with high-complexity renal tumours.

## 2. Materials and Methods

### 2.1. Study Design and Patients

A two-centre prospectively maintained database of patients undergoing renal surgery has been active at the Urology Unit, Santa Maria della Misericordia University Hospital, Udine, Italy, since 1 June 2017, and at the Oncological Urology Unit, Veneto Institute of Oncology IOV-IRCCS, Padua, Italy, since 1 November 2020. For the present study, all consecutive patients with clinically localized T1/T2 high-nephrometry-complexity tumours, defined as those with a Preoperative Aspects and Dimensions Used for an Anatomical classification (PADUA) score ≥ 10 [10] scheduled for RAPN from database inception to 31 March 2025, were extracted.

Surgeons of both centres have systematically adopted the same no-touch adaptive technique for RAPN since 16 June 2023 in the former and 1 January 2023 in the latter centre, transitioning from the same conventional technique. Patients treated with the no-touch adaptive technique (study group) were compared with their counterparts treated with the conventional one (control group).

This study was conducted in accordance with Good Clinical Practice Guidelines and the Declaration of Helsinki principles. All patients provided written informed consent and authorized data collection for scientific purposes with anonymous publication. According to Institutional Review Board regulations, retrospective observational studies using fully anonymized data from routine clinical care without identifiable biological material and involving only standard treatments are exempt from formal ethical approval.

### 2.2. Preoperative Management

Bowel preparation is not performed. A single dose of cefazolin 2 g is given intravenously approximately 20 min before trocar placement. Venous thromboembolic prophylaxis with compression stockings is used until full mobilization. Low-molecular-weight heparin is initiated on postoperative day (POD) 1 and continued for 15 days.

### 2.3. Patient Position and Port Placement

All procedures were performed by one of five expert surgeons using one of the da Vinci^®^ Si, X or Xi platforms (Intuitive Surgical, Sunnyvale, CA, USA). The surgical approach (transperitoneal or retroperitoneal) was chosen based on surgeon’s preference.

In the transperitoneal approach, the patient is placed in an extended-flank position, and a 4-arm configuration is adopted. The first 12 mm trocar is placed along the median line approximately 10 cm above the umbilicus, and is used for the AirSeal^®^ insufflation system (Conmed, Largo, FL, USA), ensuring a 12 mmHg intra-abdominal pressure. Four 8 mm robotic trocars are placed under direct vision along the pararectal line from the lower margin of the costal arch to the iliac fossa. A 12 mm laparoscopic trocar for table assistance is placed along the median line approximately 10 cm below the umbilicus. For right RAPN, a further 5 mm trocar is placed along the median line for liver retraction. Instruments are fenestrated bipolar forceps, monopolar curved scissors, and Cadiere forceps together with a 30-degree face-down endoscope and a large needle driver, if needed.

In the retroperitoneal approach, the patient is placed in a full-flank position, and a 3-arm configuration is adopted. The first 12 mm laparoscopic trocar for table assistance is placed immediately below the tip of the 11th rib. The retroperitoneal working space is then created using a dissecting balloon. Three 8 mm robotic trocars are then placed along the posterior, mid- and anterior axillary line under digital control. A 12 mm trocar used for the AirSeal^®^ system is placed immediately above the tip of the iliac crest along the mid-axillary line.

### 2.4. Surgical Technique

In the transperitoneal approach, the colon is medialized, and the renal hilum is identified. In the retroperitoneal approach, a direct access to the renal hilum is created.

The main renal artery is routinely dissected and suspended with a vessel loop. Accessory arteries, if present, are also suspended. This represents a critical safety step, given that tumour enucleation is intentionally performed in a clampless fashion and involves renal masses of high nephrometric complexity. Gerota’s fascia is then opened, and the tumour identified. A laparoscopic ultrasound probe is used to visualize endophytic tumours or exophytic tumours of larger volume or with adherent perinephric fat, and define their limits.

In the study group, no upfront arterial clamping is performed. Controlled hypotension is achieved by pharmacologically lowering systolic blood pressure to 80 to 90 mmHg with a mean arterial pressure target value ranging between 60 and 70 mmHg [11,12]. Tumour margins are marked with monopolar cautery, and a simple enucleation plan is developed with monopolar scissors, initially sharply and then bluntly, dissecting the tumour away from the peritumoral capsule along a mostly avascular plane. The feeding arteries and minor vessels identified during the enucleation are progressively and incrementally controlled with pinpoint monopolar or bipolar coagulation, or selectively secured with medium or medium–large Hem-o-lok clips. If needed, intra-abdominal pressure is temporarily increased from 12 to 18 mmHg. The table assistant maintains a clear enucleation field using one or two suction cannulas (Dolphin^®^, AB Medica S.p.A., Milan, Italy), which also provide additional stability to the kidney during the dissection. No parenchymal reconstruction is performed. Any defect of the collecting system is immediately closed with a selective Monocryl 4-0 suture, which is not considered as a renorrhaphy. Haemostasis is then checked only once the tumour is excised and the pneumoperitoneum is lowered down to 8 mmHg. Pinpoint monopolar or bipolar coagulation is applied to residual bleeders. A haemostatic agent (Hemopatch^®^ or Floseal^®^, Baxter International Inc., Deerfield, IL, USA) is then placed on the enucleation bed, and the field is checked for approximately two minutes while the systemic blood pressure is elevated to a systolic target value of 120 mmHg. In cases of persistent bleeding, selective 3-0 Monocryl sutures are placed. Only in the case of gross bleeding impeding the visualization of the dissection plane during enucleation is the main artery clamped, and selective sutures are used whenever possible, or a conventional inner single-layer renorrhaphy using 3-0 Monocryl sutures with the sliding-clip technique is conducted. The no-touch adaptive technique is showed in Supplementary Video S1.

In the control group, an on-clamp minimal enucleoresection followed by a double-layer renorrhaphy using the sliding-clip technique is performed. The inner layer approximates the medulla of the resection bed with 3-0 Monocryl interrupted sutures, and at the same time closes any defect of the collecting system. The outer layer approximates the cortex of the resection bed with 2-0 Vicryl interrupted sutures.

In both groups, Gerota’s fascia is reapproximated, and a perirenal drain is left in place.

### 2.5. Postoperative Management

An enhanced recovery pathway is followed. Postoperative pain control is achieved using intravenous non-opioid analgesics, with a gradual transition to oral paracetamol. On POD 1, oral intake is initiated, the urethral catheter is removed, and patients are encouraged to ambulate. On POD 2, the drain is removed, and henceforth patients are eligible for discharge with clear urine output and no complications.

### 2.6. Data Extraction and Study Outcomes

All data were extracted from the database by medical staff. Preoperative variables were: age at surgery, gender, body mass index, Charlson comorbidity index, American Society of Anesthesiologists (ASA) score, clinical tumour size, clinical tumour stage according to the 8th edition UICC TNM classification system, and PADUA score. Perioperative variables were: surgical approach, operating room (OR) time, estimated blood loss (EBL), red blood cell transfusion rate, warm ischemia time (WIT), and length of stay (LOS). Intraoperative complications were graded according to the EAU intraoperative adverse incident classification [13]. Conversions to intracorporeal radical nephrectomy, open radical nephrectomy or open PN were recorded.

All surgical specimens were processed according to the standard protocol, and reviewed by a dedicated uropathologist. The following parameters were assigned: tumour histological subtype according to the 5th edition WHO classification, tumour grade according to the 2012 ISUP Consensus Conference grading system [14], pathological tumour stage according to the 8th edition UICC TNM classification system, and surgical margin status.

Postoperative complications observed within 90 days from surgery were recorded and graded according to the Clavien–Dindo classification [15]. Grade 1 or 2 complications were considered minor, while grade 3 to 5 complications were classified as major. Ninety-day readmissions were also recorded. Quality criteria for accurate and comprehensive reporting of surgical outcomes were followed as recommended by the EAU guidelines [16], and are detailed in Supplementary Table S1.

Baseline and 3-month postoperative renal function was measured with the estimated glomerular filtration rate (eGFR) calculated with the 2021 Chronic Kidney Disease Epidemiology Collaboration formula [17] and stratified according to the 2023 Kidney Disease: Improving Global Outcomes classification [18]. A clinically significant renal function decline was arbitrarily defined as a ≥30% decrease in postoperative eGFR based on a proposal from a joint National Kidney Foundation and US Food and Drug Administration workshop [19].

Patients were generally followed up at 3 and 12 months in the first year, at 6-month intervals in the second year, and annually thereafter. Routine follow-up studies included a physical examination, serum creatinine, and abdominal ultrasound or computed tomography of the thorax and abdomen up to 5 years postoperatively, or when clinically indicated. For this study, only the follow-up data up to the 3-month postoperative visit were analyzed.

Study outcomes were: (1) completion rate of a fully no-touch procedure in the study group and (2) perioperative outcomes, 90-day postoperative complications, and change in 3-month renal function from baseline in the two groups. Cases in which the no-touch technique could not be fully completed—owing to the necessity of either arterial clamping, enucleoresection or renorrhaphy—were analyzed on an intention-to-treat basis.

### 2.7. Statistical Analyses

Parametric continuous variables were reported as mean ± standard deviation, whereas median and interquartile range (IQR) were used for non-parametric continuous variables. The Student *t* test, Mann–Whitney U test, and Pearson’s Chi-square test were used to compare continuous parametric, non-parametric and categorical variables, respectively, as appropriate. All clinical records were inserted in a dedicated database, and data were analyzed using SPSS v. 21.0 software (IBM Corp., Armonk, NY, USA). All reported *p* values were two-sided, and statistical significance was set at *p* < 0.05.

## 3. Results

During the study period, a total of 936 patients underwent nephrectomy for renal tumours at the two institutions: PN in 617 and RN in 319. A total of 72 patients with high-complexity renal tumours were included in this analysis, with 38 in the study and 34 in the control group. The two groups were comparable for all demographic, clinical, and pathological variables (Table 1). Of note, all patients had a single renal mass, and none had a single kidney.

In the study group, the fully no-touch technique was completed in 30/38 (79%) patients. Eight patients required adaptation. Their characteristics are detailed in Supplementary Table S2. Reasons for adaptation were: switching to main arterial clamping (*n* = 2), renorrhaphy (*n* = 5), and switching to main arterial clamping plus renorrhaphy (*n* = 1) with eventual conversion to total nephrectomy for intractable bleeding. In the three patients with a switch to main arterial clamping, the WIT was 6, 7, and 10 min. When renorrhaphy was applied, it was limited to the bleeding area and only included selective sutures in all cases but one, where a conventional single-layer suture line was applied. In the eight cases with adaptation, median EBL was 175 mL (IQR 150–213).

Perioperative outcomes for the two groups are detailed in Table 2. No intraoperative complications were observed in either group. Notably, LOS was significantly shorter in the study group.

Any-grade 90-day postoperative complications were significantly less frequent in the study (21%) vs. control (56%) group (*p* < 0.01). The major complication rate was not significantly different (*p* = 0.23). However, no single case of delayed bleeding complications sustained by intrarenal arteriovenous fistula or renal artery pseudoaneurysm was observed in the study group. Details are reported in Table 3.

A statistically significant decline in eGFR from baseline to 3-month visit was observed in the control group only (*p* = 0.01). Moreover, the proportion of patients with clinically significant eGFR decline was significantly higher in the control group (*p* = 0.02) (Table 2).

Separate perioperative and postoperative outcomes for cases with adaptation are detailed in Supplementary Table S3.

## 4. Discussion

Our study showed that the no-touch adaptive technique for RAPN is a feasible option for patients with high-complexity renal tumours, offering lower morbidity and better short-term renal function preservation compared to the conventional technique.

The transition to this technique was driven by the assumption that both ischemia and renorrhaphy may negatively impact short- and long-term renal function, with renorrhaphy potentially increasing the risk of delayed bleeding complications, such as pseudoaneurysms and arteriovenous fistulas. Additionally, we hypothesized that simple enucleation without clamping and reconstruction with incremental haemostasis would best preserve the volume and integrity of the remaining renal parenchyma, a factor known to be a key predictor of long-term renal function after partial nephrectomy [20].

In fact, the individual impacts of each of the three main surgical steps of the RAPN procedure, i.e., arterial inflow control, tumour excision and parenchymal reconstruction, remain to be conclusively determined. A recent systematic review with quantitative analysis revealed no clinically relevant differences between off-clamp and on-clamp RAPN in perioperative outcomes, such as complication rate, EBL, and blood transfusion rate, and functional outcomes, such as the percentage decline in eGFR [21]. In another systematic review, a pooled analysis revealed an advantage for tumour enucleation over standard resection in terms of avoidance of renal artery clamping, overall and major complications, length of stay, and renal function during RAPN [22]. As for parenchymal reconstruction, two systematic reviews comparing outcomes with sutureless versus standard double-layer renorrhaphy during RAPN showed the advantage of sutureless renorrhaphy in terms of reduced OR time and WIT, and reduced decline in eGFR, with no significant difference in the EBL and complication rate [23,24]. Another review concluded with different, even contrasting, findings [25]. The uncertainty around current evidence arises due to significant variability across studies, including differences in preoperative patient characteristics, tumour nephrometry complexity, inconsistent reporting of tumour excision techniques, and variations in the methods and timing used to assess renal function. Additionally, surgeon experience—a factor that may critically influence functional outcomes—has been infrequently considered in comparative studies [26,27].

Several findings of our analysis are noteworthy. First, a fully no-touch procedure was achieved in most patients. In the remaining cases, the adaptive nature of the technique allowed for the selective use of conventional maneuvers, such as arterial clamping, enucleoresection, and/or renorrhaphy, thus providing intraoperative flexibility tailored to individual circumstances. Importantly, these cases should not be considered directly comparable to those treated with the conventional approach, as clamping, when required, was applied only for a limited portion of the procedure, and renorrhaphy, when necessary, was restricted to selective sutures (typically one or two) in virtually all cases.

Second, in contrast to most reports in the literature, the use of an off-clamp technique under controlled systemic arterial hypotension, combined with incremental coagulation of the resection field, resulted in limited EBL without the need for intra- or postoperative blood transfusions. These outcomes are comparable, or even superior, to those reported in most contemporary on-clamp RAPN series [28,29], and suggest that monopolar and bipolar coagulation of renal vessels can be highly effective, thus challenging long-standing assumptions.

Third, no single case of delayed renal bleeding due to pseudoaneurysms or arteriovenous fistulas was observed, which may be attributable to the omission of conventional single- or double-layer renorrhaphy and the use of on-demand, selective and more superficial parenchymal sutures for bleeding control only. CT imaging was only obtained when bleeding prompted evaluation; therefore detection of asymptomatic pseudoaneurysms might have been missed. However, we believe imaging all cases after surgery is neither feasible nor cost-effective on a routine basis.

Fourth, we observed that avoiding global renal ischemia facilitates the initial identification of the dissection plane for simple tumour enucleation, enhancing the visibility of the pseudocapsule boundary. In addition, the use of an incremental and pinpoint haemostasis strategy ensures good visualization of the enucleation field, while reducing the likelihood of violating the collecting system, thereby potentially lowering the risk of urinary fistula. All these aspects are particularly valuable in the management of high-complexity tumours, due to their generally large contact surface area.

Fifth, a beneficial effect of the no-touch adaptive technique on early renal function preservation could be demonstrated. Due to the small sample size, we could not analyze the findings after stratification by baseline renal function, although we hypothesize that this effect might be more evident in patients with impaired baseline renal function, who should be the focus of future research. Moreover, nuclear renography with split renal function might have provided more granular data in this regard.

To the best of our knowledge, the combination of an off-clamp and sutureless approach with simple enucleation has only been reported in a single recent comparative study. In a propensity score-matched analysis including a total of 160 patients, Brassetti et al. retrospectively compared the outcomes of sutureless versus single-layer renorrhaphy off-clamp RAPN with simple enucleation [30]. Patients in the sutureless group had shorter LOS (2 vs. 3 days, *p* < 0.001) and a higher likelihood of achieving trifecta (96% vs. 84%, *p* = 0.008), according to their own definition [31]. Additionally, the rates of grade ≥ 3 complications (2% vs. 5%, *p* = 0.12), blood transfusions (3% vs. 4%, *p* = 0.62), and urinary fistulas (2% vs. 1%, *p* = 0.18) were comparable in the two groups. However, the proportion of patients with high-nephrometry-complexity tumours was low (only 11 cases in the propensity-adjusted cohort), and no stratified outcomes by tumour complexity were reported. The major difference in the technique is the type of haemostasis, whereby the Authors applied a continuous and extensive coagulation to the resection bed with high-energy monopolar cautery after tumour removal, until a firm uniform eschar was formed. We, instead, applied an incremental and pinpoint haemostasis with both monopolar and bipolar coagulation during the entire tumour excision. Our approach also minimized the haemostatic thermal injury to the collecting system, thus potentially reducing the risk of delayed urinary fistulas.

Finally, whilst reiterating the key role of surgeon experience, we think the no-touch approach for RAPN does not inherently introduce a higher technical complexity compared with the conventional technique. Rather than relying on novel instruments or unfamiliar oncological principles, it reorganizes well-established procedural steps into a graded and adaptive workflow, allowing escalation to arterial clamping or renorrhaphy only when required. Importantly, the absence of upfront warm ischemia may eliminate time pressure during tumour excision, and may facilitate identification of the enucleation plane, thus improving intraoperative visualization and decision-making. A recent study conducted at two different institutions showed that sutureless off-clamp RAPN is a feasible and safe nephron-sparing technique, even when performed by a novice robotic surgeon [32]. Nevertheless, structured mentorship, appropriate case selection, and progressive autonomy remain essential, particularly during the early learning phase. Prospective multicentre studies specifically addressing learning curves, surgeon experience, and reproducibility across different practice settings are warranted before broad generalization of these findings can be advocated.

Our study is not devoid of limitations. First, this is a retrospective unmatched comparison of two temporally distinct cohorts of patients, all treated by trained surgeons at two high-volume tertiary referral centres, with the inherent shortcomings of selection and chronological bias. Nonetheless, robotic technology, perioperative care pathways and institutional protocols remained unchanged over the entire study period. Moreover, not all the surgeons included in the analysis performed both techniques, but all qualified as experts. Second, caution should be exerted when interpreting certain subgroup analyses due to the relatively limited sample size, which did not allow for multivariable adjustment by patient-, tumour-, and surgeon-related factors. In particular, major uncommon complications might have been underreported. Nevertheless, the cohort of analyzed patients, i.e., those with high-nephrometry-complexity tumours, sits among the largest of those reported in the literature where a comparative analysis of surgical techniques has been performed. Third, the generalisability of our findings to the broad category of high-nephrometry-complexity tumours is arguable, since we excluded cases treated with RN. Consequently, the included cases may reflect a more favourable surgical profile in terms of technical feasibility and complications. Fourth, further evaluation of the functional and oncological outcomes in this high-risk patient population is necessary before implementing this technique on a larger scale. Of particular interest would be the data on recurrence-free survival for those cases with positive surgical margins, and those on medium-to-long-term renal function, especially for patients with significant eGFR decline after surgery, where the early benefit might diminish with a longer observation time.

## 5. Conclusions

In this comparative analysis of patients with high-complexity renal tumours, the no-touch adaptive technique for RAPN proved to be feasible and reproducible, with a high rate of completion without the need for conversion to conventional steps. This approach was associated with a significant reduction in overall postoperative complications, shorter length of hospital stay, and improved early preservation of renal function, with no cases of clinically meaningful eGFR decline observed at 3 months postoperatively. These findings support the underlying surgical rationale of minimizing renal ischemia and avoiding extensive parenchymal reconstruction whenever safely possible, particularly in technically challenging cases where the balance between oncological control and functional preservation is critical.

Despite these encouraging results, the present study has limitations that warrant careful interpretation. Its retrospective design, the relatively small sample size, and the comparison of temporally distinct cohorts may introduce selection and chronological biases. In addition, the short-term follow-up precludes any definitive conclusions regarding long-term renal functional outcomes and oncological safety. Future research should focus on adequately powered prospective, ideally randomized, multicentre, and multisurgeon studies to validate these findings, assess reproducibility across different surgical settings and levels of expertise, and evaluate long-term functional and oncological endpoints. In this context, the no-touch adaptive technique represents a promising step toward a more tailored and function-preserving surgical strategy in RAPN.

## Figures and Tables

**Table 1:** Demographic, preoperative, and pathological characteristics of the two groups of patients with high-complexity renal tumours scheduled for robot-assisted partial nephrectomy and included in the comparative analysis.

Variables	Total Cases (*n* = 72)	Study Group (*n* = 38)	Control Group (*n* = 34)	*p* Value
Age, years, median (IQR)	65	64.5	65	0.45
	(60–69)	(57–69.8)	(57.5–70)	
Male gender, *n* (%)	43 (60)	20 (53)	23 (68)	0.36
BMI, kg/m^2^, median (IQR)	27.7	27.5	28	0.45
	(24.6–31.5)	(24.5–31)	(25–32)	
Charlson comorbidity index > 2, *n* (%)	18 (25)	10 (26)	8 (24)	0.42
ASA score, *n* (%)				0.70
- 1	22 (31)	11 (29)	11 (32)	
- 2	29 (40)	16 (42)	13 (38)	
- 3	21 (29)	11 (29)	10 (29)	
Clinical tumour size, cm, median (IQR)	45	49	44	0.21
	(41–52)	(40.5–54.5)	(38–45)	
Right-side tumour, *n* (%)	33 (46)	17 (45)	16 (47)	0.88
Clinical tumour stage, *n* (%)				0.19
- T1a	22 (31)	10 (26)	12 (35)	
- T1b	46 (64)	25 (66)	21 (62)	
- T2a	4 (6)	3 (8)	1 (3)	
PADUA score, *n* (%)				0.11
- 10	41 (57)	21 (55)	20 (59)	
- 11	19 (26)	10 (26)	9 (26)	
- 12	11 (15)	6 (16)	5 (15)	
- 13	1 (1)	1 (3)	0 (0)	
Baseline eGFR, mL/min/1.73 m^2^, median (IQR)	80.5	81	80	0.15
	(68–89.5)	(68.3–90.5)	(68–88.5)	
Baseline CKD stage, *n* (%)				0.57
- 1	24 (33)	13 (34)	11 (32)	
- 2	40 (56)	21 (55)	19 (56)	
- 3A	6 (8)	3 (8)	3 (9)	
- 3B	2 (3)	1 (3)	1 (3)	
Pathological tumour stage, *n* (%)				0.33
- T1a	21 (29)	9 (24)	12 (35)	
- T1b	32 (44)	24 (63)	20 (59)	
- T2a	31 (43)	3 (8)	1 (3)	
- T3a	16 (22)	2 (5)	1 (3)	
Tumour histological subtype, *n* (%)				0.18
- Clear cell RCC	62 (86)	32 (84)	30 (88)	
- Non-clear cell RCC	6 (8)	4 (11)	2 (6)	
- Benign	4 (6)	2 (5)	2 (6)	
Tumour grade, *n* (%)				0.36
- 1	13 (18)	6 (16)	7 (21)	
- 2	46 (64)	24 (63)	22 (65)	
- 3	10 (14)	6 (16)	3 (9)	
- NA	4 (6)	2 (5)	2 (6)	
Positive surgical margins, *n* (%)	3 (4)	2 (5)	1 (3)	0.41

ASA: American Society of Anesthesiologists; BMI: body mass index; CKD: chronic kidney disease; eGFR: estimated glomerular filtration rate; IQR: interquartile range; NA: not applicable; PADUA: Preoperative Aspects and Dimensions Used for an Anatomical classification; RCC: renal cell carcinoma.

**Table 2:** Perioperative and postoperative outcomes of the two groups of patients with high-complexity renal tumours scheduled for robot-assisted partial nephrectomy and included in the comparative analysis.

Variables	Total Cases (*n* = 72)	Study Group (*n* = 38)	Control Group (*n* = 34)	*p* Value
Transperitoneal approach, *n* (%)	67 (93)	35 (92)	32 (94)	1.00
Operating room time, min, median (IQR)	135	125	141	0.19
	(110–240)	(112–225)	(125–230)	
Estimated blood loss, mL, median (IQR)	175	150	180	0.43
	(95–360)	(75–250)	(100–400)	
Perioperative blood transfusions, *n* (%)	3 (4)	0 (0)	3 (9)	0.09
Length of stay, days, median (IQR)	4	3	5	<0.001
	(4–7)	(3–4)	(5–7)	
90-day readmissions, *n* (%)	2 (3)	0 (0)	2 (6)	0.23
3-month eGFR, mL/min/1.73 m^2^, median (IQR)	75.5	79	70.5	0.01
	(64.3–83.7)	(69.5–89)	(60–80.7)	
3-month CKD stage, *n* (%)				0.50
- 1	22 (31)	13 (34)	9 (26)	
- 2	36 (50)	20 (53)	16 (47)	
- 3A	10 (14)	4 (11)	6 (18)	
- 3B	4 (6)	1 (3)	3 (9)	
3-month CKD stage progression, *n* (%)	8 (11)	1 (3)	7 (21)	0.02
3-month eGFR decline ≥ 30%, *n* (%)	5 (7)	0 (0)	5 (15)	0.02

CKD: chronic kidney disease; eGFR: estimated glomerular filtration rate; IQR: interquartile range.

**Table 3:** Worst single 90-day postoperative complications scored according to Clavien–Dindo classification in the two groups of patients with high-complexity renal tumours scheduled for robot-assisted partial nephrectomy and included in the comparative analysis.

Grade	Total Cases (*n* = 72), *n* (%)	Study Group (*n* = 38), *n* (%)		Control Group (*n* = 34), *n* (%)		Complication Type	Treatment
1	11 (15)	5 (13)	2	6 (18)	3	Nausea/Vomit	Antiemetics
			1		0	Perirenal haematoma	Analgesics
			2		3	Fever of unknown origin	Antipyretics
2	9 (13)	1 (3)	1	8 (24)	3	Fever of unknown origin	Antipyretics and antimicrobials
			0		2	Perirenal haematoma with anaemia	Blood transfusions
			0		1	Arterial hypotension with anaemia	Blood transfusions
			0		1	Atrial fibrillation	Antiarrhythmics
			0		1	Pulmonary embolism	Anticoagulants
3a	7 (10)	2 (5)	2	5 (15)	2	Calyceal urinary leakage	Retrograde ureteric catheter placement
			0		2	Intrarenal arteriovenous fistula	Transcatheter arterial embolization
			0		1	Renal artery pseudoaneurysm	Transcatheter arterial embolization

## Data Availability

The data that support the findings of this study are available from the corresponding author upon reasonable request.

## Supplementary Materials

The following supporting information can be downloaded at https://www.mdpi.com/article/10.3390/cancers18101577/s1, Table S1: Quality criteria for accurate and comprehensive reporting of surgical outcomes recommended by the European Association of Urology Guidelines; Table S2: Demographic, preoperative, and pathological characteristics of the eight patients with high-complexity renal tumours scheduled for robot-assisted partial nephrectomy in whom the no-touch technique required adaptation (arterial clamping, enucleoresection and/or renorrhaphy); Table S3: Perioperative and postoperative outcomes of the eight patients with high-complexity renal tumours scheduled for robot-assisted partial nephrectomy in whom the no-touch technique required adaptation (arterial clamping, enucleoresection and/or renorrhaphy); Video S1: Videoclip showing the main features of the no-touch adaptive technique for robot-assisted partial nephrectomy.
