# Supplementary material for: No-Touch Adaptive Versus Conventional Robot-Assisted Partial Nephrectomy for Localized Renal Tumours with High Nephrometry Complexity: A Comparative Analysis of Early Outcomes
Source: Cancers (Basel). 2026 May 12;18(10):1577. doi: 10.3390/cancers18101577 (PMC13204421; doi:10.3390/cancers18101577)
Supplement: Supplementary file 1 [file cancers-18-01577-s001.zip › Supplementary_Table_S2_R1.pdf]

Supplementary Table S2. Demographic, preoperative, and pathological characteristics of the eight patients with high-complexity renal tumours scheduled for robot-assisted partial nephrectomy in whom the *no-touch* technique required adaptation (arterial clamping, enucleoresection and/or renorrhaphy).

**Variables**	**Study subgroup (n=8)**
Age, years, median (IQR)	66.5 (64.3 - 72.5)
Male gender, n (%)	6 (75)
BMI, kg/m^2, median (IQR)	26.5 (25 - 27.2)
Charlson comorbidity index >2, n (%)	3 (38)
ASA score, n (%)	
- 1	1 (13)
- 2	4 (50)
- 3	3 (38)
Clinical tumour size, mm, median (IQR)	55 (50 - 63)
Right side tumour, n (%)	3 (38)
Clinical tumour stage, n (%)	
- T1a	1 (13)
- T1b	6 (75)
- T2a	1 (13)
PADUA score, n (%)	
- 10	1 (13)
- 11	6 (75)
- 12	1 (13)
Baseline eGFR, ml/min/1.73 m^2, median (IQR)	77.5 (69.8 - 84.5)
Baseline CKD stage, n (%)	
- 1	2 (25)
- 2	6 (75)
Pathological tumour stage, n (%)	
- T1a	1 (13)
- T1b	6 (75)
- T2a	1 (13)
Tumour histological subtype, n (%)	
- clear cell RCC	6 (75)
- non-clear cell RCC	1 (13)
- benign	1 (13)
Tumour grade, n (%)	
- 1	1 (13)
- 2	3 (38)
- 3	3 (38)
- NA	1 (13)
Positive surgical margins, n (%)	1 (13)

ASA: American Society of Anesthesiologists; BMI: body mass index; CKD: chronic kidney disease; eGFR: estimated glomerular filtration rate; IQR: interquartile range; NA: not applicable; PADUA: Preoperative Aspects and Dimensions Used for an Anatomical classification; RCC: renal cell carcinoma
