# Supplementary material for: No-Touch Adaptive Versus Conventional Robot-Assisted Partial Nephrectomy for Localized Renal Tumours with High Nephrometry Complexity: A Comparative Analysis of Early Outcomes
Source: Cancers (Basel). 2026 May 12;18(10):1577. doi: 10.3390/cancers18101577 (PMC13204421; doi:10.3390/cancers18101577)
Supplement: Supplementary file 1 [file cancers-18-01577-s001.zip › Supplementary_Table_S3_R2.pdf]

Supplementary Table S3. Perioperative and postoperative outcomes of the eight patients with high-complexity renal tumours scheduled for robot-assisted partial nephrectomy in whom the *no-touch* technique required adaptation (arterial clamping, enucleoresection and/or renorrhaphy).

**Variables**	**Study subgroup (n=8)**
Transperitoneal approach, n (%)	7 (88)
Operating room time, min, median (IQR)	122.5 (117.5 - 135)
Estimated blood loss, ml, median (IQR)	175 (150 - 213)
Perioperative blood transfusions, n (%)	0 (0)
Length of stay, days, median (IQR)	3 (3 - 3.5)
90-day readmissions, n (%)	0 (0)
90-day complications, n (%)	3 (38)*
3-month eGFR, ml/min/1.73 m^2, median (IQR)	77 (75-90)
3-month CKD stage, n (%)	
- 1	2 (25)
- 2	5 (63)
- 3A	1 (13)
3-month CKD stage progression, n (%)	1 (13)
3-month eGFR decline ≥30%, n (%)	0 (0)

CKD: chronic kidney disease; eGFR: estimated glomerular filtration rate; IQR: interquartile range
* fever of unknown origin in 2, calyceal urinary leakage in 1
